# From Cure to Crisis: Understanding the Evolution of Antibiotic-Resistant Bacteria in Human Microbiota

**DOI:** 10.3390/biom15010093

**Published:** 2025-01-09

**Authors:** Hamed Tahmasebi, Neda Arjmand, Marzieh Monemi, Ali Babaeizad, Farnaz Alibabaei, Negar Alibabaei, Aisa Bahar, Valentyn Oksenych, Majid Eslami

**Affiliations:** 1 School of Medicine, Shahroud University of Medical Sciences, Shahroud 36147-73943, Iran; 2 Department of Obstetrics and Gynecology, Tehran Medical University, Tehran 14167-53955, Iran; 3 Department of Basic Science, Faculty of Pharmacy and Pharmaceutical Science, Tehran Medical Science, Islamic Azad University, Tehran 19395-1495, Iran; 4 Student Research Committee, Semnan University of Medical Sciences, Semnan 35147-99442, Iran; 5 Student Research Committee, Mazandaran University of Medical Sciences, Sari 48157-33971, Iran; 6 Department of Biochemistry, Semnan University of Medical Sciences, Semnan 35147-99442, Iran; 7 Faculty of Medicine, University of Bergen, 5020 Bergen, Norway; 8 Cancer Research Center, Semnan University of Medical Sciences, Semnan 35147-99442, Iran; 9 Department of Bacteriology and Virology, Faculty of Medicine, Semnan University of Medical Sciences, Semnan 35147-99442, Iran

**Keywords:** antibiotic-resistant, crisis, evolution, hospital-acquired infections, microbiota

## Abstract

The growing prevalence of antibiotic-resistant bacteria within the human microbiome has become a pressing global health crisis. While antibiotics have revolutionized medicine by significantly reducing mortality and enabling advanced medical interventions, their misuse and overuse have led to the emergence of resistant bacterial strains. Key resistance mechanisms include genetic mutations, horizontal gene transfer, and biofilm formation, with the human microbiota acting as a reservoir for antibiotic resistance genes (ARGs). Industrialization and environmental factors have exacerbated this issue, contributing to a rise in infections with multidrug-resistant (MDR) bacteria, such as methicillin-resistant *Staphylococcus aureus* (MRSA) and carbapenem-resistant *Enterobacteriaceae*. These resistant pathogens compromise the effectiveness of essential treatments like surgical prophylaxis and chemotherapy, increase healthcare costs, and prolong hospital stays. This crisis highlights the need for a global One-Health approach, particularly in regions with weak regulatory frameworks. Innovative strategies, including next-generation sequencing (NGS) technologies, offer promising avenues for mitigating resistance. Addressing this challenge requires coordinated efforts, encompassing research, policymaking, public education, and antibiotic stewardship, to safeguard current antibiotics and foster the development of new therapeutic solutions. An integrated, multidimensional strategy is essential to tackle this escalating problem and ensure the sustainability of effective antimicrobial treatments.

## 1. Historical Overview of Antibiotic Use

In the early 20th century, the discovery of antibiotics was a humongous turning point in the world of medicine. The root of the term “antibiotic” goes back to ancient Greek, and its literal meaning is “against life”, as antibiotics are utilized to kill bacteria [1]. Their true history also goes back to ancient times, even before the term of antibiotic originated, when natural extracts of some molds and plants were used for their healing features [2,3]. Later, an American microbiologist called Selman Waksman, along with his team, introduced the concept of antibiotics by succeeding in separating chemical substances from some microorganisms that could prevent the growth of other microbes. Ultimately, the discovery of penicillin by Alexander Fleming in 1928 is known as the starting point of novel antibiotic therapy, which linked ancient knowledge of using mold against infections to the modern era [4,5]. Within the following decades, the development of new classes of antibiotics, like chloramphenicol, cephalosporins, erythromycin, vancomycin, and others, and their widespread use to treat fatal infections, have been marked as huge progress [4,6]. During the Second World War, penicillin was widely used, especially among troops, in order to treat infected wounds. Meanwhile, its efficacy against gonorrhea and syphilis boosted its usage in military units to prevent sexually transmitted diseases [3]. Antibiotics like amoxicillin and quinolones were also contributory post-World War II, with their effects on a broader spectrum of infections. With the innovation of daptomycin, third-generation cephalosporins, and linezolid to combat Gram-negative bacteria, the development of antibiotics continued, and made surgical operations and organ transplants safer by preventing post-surgery infections [7]. The introduction of antibiotics also decreased infant and maternal mortality rate remarkably by aiding the treatment of infections during pregnancy [5].

Cephalosporins were first discovered in 1945, extracted from *Acremonium* mold to function as a bactericidal antibiotic; then, in 1946, streptomycin was released to the market for the treatment of tuberculous meningitis and tuberculosis. This antibiotic, isolated as a product of *Streptomyces griseus*, can inhibit protein synthesis. Chloramphenicol is another protein synthesis-inhibiting antibiotic that was refined from *Streptomyces venezuelae* in 1947, and due to its profound permeability in tissues, was found to be effective for a broad spectrum of infections. However, because of the numerous toxicities reported in 1960, it is now rarely prescribed. The discovery of macrolides started with erythromycin, which was produced from *Saccharopolyspora erythraea* in 1952, and they are now the second most-used and -prescribed antibiotics. One of the most essential antibiotics is vancomycin, which still counts as a last-resort for resistant infections. It was discovered as a glycopeptide product of *Amycolatopsis orientalis* in 1956, and has the ability to interrupt the synthesis of bacterial cell walls [3,8,9].

Antibiotics can be categorized based on their mechanism of action; some exert their effect by disrupting the synthesis of bacterial cell walls, some cause impairments in protein or nucleic acid production, and some may disrupt folate metabolism. Another way to classify antibiotics is based on the spectrum of infections against which they are functional and effective; for instance, antibiotics of the broad-spectrum category are practical for infections caused by both Gram^+^ and Gram^-^ groups [3]

As advantageous as they are in clinical practice, over time, with the overuse of antibiotics, antibiotic resistance has become an alarming concern. The ability of pathogens to survive against antibiotics that threaten their existence and restrain their proliferation is called antibiotic resistance [10]. Penicillinase, which is able to dissolve penicillin, was first identified in a bacterium in 1940; it was the first reported enzyme able to destroy the structure of an antibiotic drug. In the following years, more research reported the presence of this deactivating enzyme in some other microbes, and over the subsequent decades, the exposure of bacterial populations to a high concentration of different antibiotics exacerbated the resistance situation [11,12,13].

**Figure 1:**
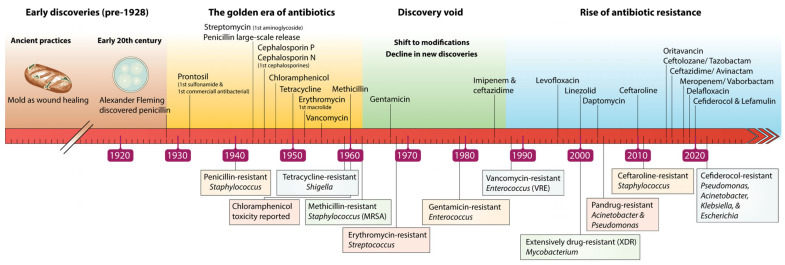
Significant events in antibiotic evolution, including the discovery of new antibiotics and the emergence of resistance to various types.

This issue needs to be addressed publicly in order to raise awareness about antibiotic resistance, and the overuse of antibiotics should be reassessed, because the consequences are irreparable. For instance, infections that were once easily managed with standard antibiotic treatments have now developed resistance, making them significantly more challenging to treat. This growing resistance has resulted in prolonged hospital stays for affected patients, increasing the risk of severe complications and mortality [4,5]. Due to the severity of the problem, the World Health Organization (WHO) released a list of the 12 most health-threatening families of bacteria in 2017, which included *Pseudomonas*, *Acinetobacter*, *E. coli*, *K. pneumoniae*, etc. These families were reported as multidrug-resistant (MDR) pathogens with a great risk of causing severe infections in the bloodstream and respiratory system [12,14]. However, developing a new antibiotic is a long process, and pharmaceutical companies are reluctant to invest because of the poor profitability and protractedness of the process [4] Figure 1.

## 2. Mechanisms of Antibiotic Resistance: Genetic Mutations and Their Implications in Bacterial Evolution

**Figure 2:**
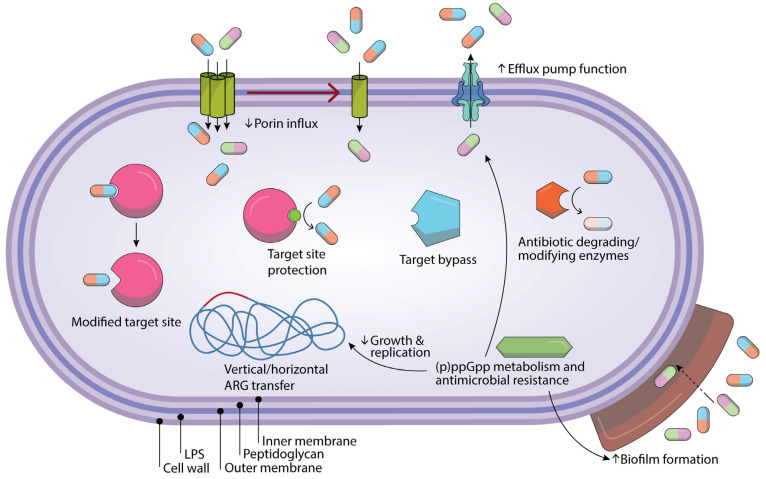
This figure illustrates the various mechanisms through which bacteria can develop resistance to antibiotics.

The first report regarding antibiotic resistance was produced back in 1930, when sulfonamides were first introduced. At the time, this predicted the inevitable occurrence of antibiotic resistance, despite the presence of fatal pathogens. Microbial strains obtain resistance genes from another bacterium via transgenes, phages, or plasmids, or through instability and mutations in chromatin, all of which lead to cross-resistance. Over the years, bacteria have utilized different ways to evade the various mechanisms of action executed by different classes of antibiotics, such as interfering with protein synthesis, dissolving the components of bacterial cell walls, and disrupting the metabolic pathways or nucleic acid production [15]. One resistance mechanism is genetic mutation, forming a “resistome” that leads to primary resistance without drug exposure; this acquired resistance, resulting from DNA mutations, is transferred through the vertical pathway [4,12]. These mutations can happen through addition, deletion, inversion, substitution, or duplication, and be easily transferred to the next generation; for instance, mutations in the DNA gyrase coding genes *gyrA* and *gyrB* can result in resistance against fluoroquinolones, or resistance to macrolides can be caused by mutations in ribosomal RNA 23S sequences [16]. Plasmid transfer is another mechanism by which resistance-containing genes are transferred to another bacterium and its offspring. These mutations have the ability to change the target site’s structure, which reduces the antibiotic’s affinity and effectiveness. The inhibition of *Mycobacterium tuberculosis*, for instance, is caused by mutations in the RNA polymerase gene, which is the binding site for the antibiotic rifampicin [4]. Another significant consequence of these mutations is the upregulation of efflux pumps, which are specialized protein transporters capable of actively expelling antibiotics from the cell. This mechanism prevents antibiotics from reaching their intracellular targets, thereby reducing their efficacy and contributing to the development of antimicrobial resistance. MexAB-OprM is an example of efflux pump in *Pseudomonas aeruginosa* (*P. aeruginosa*) that can pump out aminoglycosides and fluoroquinolones [4,17]. Also, there are an abundant number of efflux pumps in *Acinetobacter baumannii* that enable resistance to tetracyclines, chloramphenicol, aminoglycosides, fluoroquinolones, trimethoprim, and different β-lactams [12] Figure 2.

Lateral or horizontal gene transfer is another method by which bacteria develop acquired resistance to antibiotics. The precise example that can be named here is the rapid spread of the *blaNDM-1* gene in *Enterobacteriaceae*. This gene encodes a carbapenemase enzyme that further diminishes the effectiveness of carbapenem, a potentially powerful antibiotic [17]. Horizontal gene transfer occurs through three primary mechanisms: conjugation, transduction, and transformation [4]. Conjugation, in particular, is often facilitated under stressful conditions, such as exposure to antibiotics or heavy metals, which can drive the transfer of genetic material between bacteria [17].

**Figure 3:**
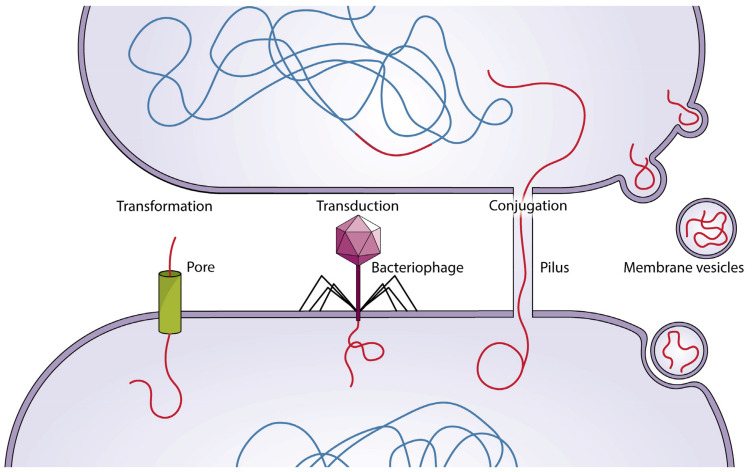
Horizontal gene transfer in bacteria occurs through four main mechanisms: transformation (the uptake of free DNA from the environment), transduction (DNA transfer via bacteriophages), conjugation (DNA transfer through direct contact and plasmids), and membrane vesicles (transfer of genetic material via vesicles, such as vancomycin resistance genes). These processes play a key role in genetic diversity and the spread of antibiotic resistance.

In conjugation, DNA is shifted from a cell to another via adhesions on the cell surface or pili. The whole process is facilitated by conjugative machinery that is encoded by plasmid genes or the conjugative components in the chromosome. An example of this is extended-spectrum β-lactam resistance genes on plasmids spreading through the *Enterobacteriaceae* and *pseudomonas* families by conjugation [18]. In the transduction method, bacteriophages are involved. By transferring beneficial genes to another host, they can spread and boost their own survival as well. An observation of extracted bacteriophages from hospital-acquired MRSA infections has shown the successful transduction of antibiotic-resistant genes (ARGs) to vulnerable strains [19]. In both conjugation and transduction, plasmids are involved. Plasmids that are circular extrachromosomal DNA molecules can be transferred either via direct contact between bacterial cells in conjugation, or via bacteriophages in the transduction method [17]. In the latter method, transformation happens when the bacteria take up extracellular DNA fragments, leading them to be functionally expressed. Research has indicated that *Helicobacter pylori* is able to experience transformation and obtain genes through natural competence, and eventually turn into a virulent pathogen [20] (Figure 3).

Sometimes, bacteria tolerate challenging conditions by altering their metabolic state. During the stationary phase, they can produce biofilm, which is another mechanism of resistance against antibiotics. Biofilms are organized communities of bacteria, placed in an extracellular matrix, that allow them to live on surfaces and impede antibiotic from penetrating and gaining access to bacteria; all in all, biofilms keeps bacteria safe and away from drugs and the immune system. Inside the biofilm, bacteria are able to communicate and match their resistance mechanisms. *P. aeruginosa* is a classic example of a bacterium that forms biofilms in the airways of patients with cystic fibrosis. This bacterium can regulate the expression of superoxide dismutase and catalase, and eventually raise resistance against hydrogen peroxides. Also, the biofilm prevents the antibiotic from reaching the bacterial cell. Altogether, this leads to infection persistence and a chronic and recurrent state of cystic fibrosis [4].

Another interfering factor in antibiotic resistance in bacteria is the expression of the molecule (p) ppGpp. This mechanism represents a sophisticated adaptive strategy employed by bacteria in response to environmental stressors, such as exposure to antibiotics or nutrient deprivation. In such conditions, bacteria synthesize this molecule, and then the elevated level of (p) ppGpp leads to stress adaptation and the activation of survival mode. The bacteria prioritize the conservation of energy, so they downregulate replication and growth and start forming biofilms. By decreasing their growth rate, the bacteria become less vulnerable to antibiotics, since many antibiotics target dividing cells. Lastly, some bacteria can circumvent the mechanism of action of antibiotics in other ways. For instance, in the case of trimethoprim, bacteria may utilize an alternative enzyme instead of dihydrofolate reductase, which is the primary target of trimethoprim, thereby rendering the antibiotic ineffective and futile [4]. It is important to note that sometimes, the resistance of some bacterial species is unrelated to previous antibiotic exposure; for example, *Escherichia coli* (*E. coli*) is naturally resistant to vancomycin. This type of resistance is called intrinsic resistance, which is classified in a different category to acquired resistance [12].

### 2.1. Carbapenem-Resistant Enterobacteriaceae (CRE)

Antibiotic-resistant bacteria are facing a more recent challenge with the presence of carbapenemases. Resistance to carbapenem antibiotics is commonly caused by β-lactamases carried on plasmids. *OXA65*, *NDM*, *IMP*, *VIM*, and *KPC* are the most prevalent variants, with minimal occurrence in Western Europe [21,22]. Hospital admissions in countries like India and Pakistan, where carbapenemases are prevalent, are often associated with infections from these isolates. The increasing occurrence of carbapenemases is a cause for concern because of the limited availability of other treatment options and the near absence of new antibiotics in development. An increase in the prevalence of isolates that produce carbapenemase could result in a situation comparable to the era before antibiotics, similar to the time prior to the discovery of penicillin [23,24].

Despite this, resistance to carbapenems has been associated with specific ESBLs such as *OXA-40*, *OXA-48*, and *OXA-23*. In the decade from 2000 to 2010, there was a 3% increase in Carbapenem-resistant *Enterobacteriaceae* (CRE) infections in the USA, resulting in mortality rates reaching up to 48% because of limited treatment choices [25]. The clinical significance of mobile carbapenemases lies in their unique capacity to break down the β-lactam ring in carbapenems and their tendency to disseminate, making carbapenem resistance a crucial issue. In these instances, colistin may be used as a treatment, although resistance to this medication is spreading rapidly [26]. Previously, it was assumed that colistin resistance was exclusively associated with alterations in chromosomal DNA. That being said, it was in 2015 that the *mcr-1* gene, associated with colistin resistance, was first recognized. In China, *E. coli* isolates revealed the presence of *MCR-1*, an enzyme that modifies the colistin target, causing a reduction in drug efficacy [27]. There are currently eight known mcr variations, with at least two having a worldwide presence. Consequently, the range of available treatments is greatly restricted, and the increasing resistance to colistin in infections that are resistant to carbapenem is a major worry for worldwide health. The development and international spread of mobile carbapenemases and *mcr* genes in clinical pathogen samples emphasize the scale and severity of the horizontal transfer of antibiotic resistance genes [28,29,30].

### 2.2. Spread of MRSA

Shortly after penicillin was first discovered, the initial strain of bacteria resistant to the antibiotic was identified. Penicillin resistance was widespread, reaching about 80%, when methicillin was first introduced in 1959, and has persisted at high levels ever since [31]. In Europe, a strain of *S. aureus* resistant to methicillin (MRSA) emerged shortly after the drug was introduced. MRSA has become more common and has expanded globally as time has passed. The emergence of MRSA is not confirmed, but it is suggested that SCCmec has been integrated into multiple *S. aureus* lineages (multi-clone theory) instead of being acquired by a single strain that was the precursor to all MRSA clones [31,32]. Various typing techniques have revealed that numerous MRSA strains are widespread worldwide, with many of these strains also being found in the Netherlands, Belgium, and Germany [31,32,33,34]. The most frequently encountered strains include ST22-MRSA-IV (EMRSA-15), ST5-MRSA-II (New York/Japan or Rhine Hesse clone), ST45-MRSA-IV (Berlin clone), ST5-MRSA-IV (pediatric clone), and ST8-MRSA-IV (EMRSA-2/6 or USA300 if PVL-positive). The occurrence of MRSA varies both within and among countries and patient cohorts. MRSA is more commonly found in hospitals and nursing homes than in the community at large [31].

### 2.3. Beta-Lactamases

Gram-positive bacteria, much like *S. aureus*, have developed resistance mechanisms against antibiotics over time. These mechanisms began emerging well before the discovery of penicillin. For instance, *E. coli* strains capable of producing penicillinase, a type of β-lactamase, were identified early on, and this ability spread rapidly to other bacterial species [35,36]. The introduction of cephalosporins in clinical practice initially expanded treatment options. However, this development was soon accompanied by the rise of more potent β-lactamases, such as *TEM* and *SHV*, which were capable of breaking down early cephalosporins, significantly reducing their effectiveness. By the early 1970s, the prevalence of β-lactamase-producing *E. coli* isolates became widespread. This phenomenon continued with the advent of β-lactamase-resistant cephalosporins, particularly third-generation cephalosporins. The increased use of these antibiotics led to the identification of Extended-Spectrum Beta-Lactamases (ESBLs), a more potent class of β-lactamases capable of hydrolyzing a broader spectrum of β-lactam antibiotics, including third-generation cephalosporins [22,37]. As the use of these cephalosporins grew, the emergence of new, more effective β-lactamases, such as ESBLs, became more prominent, and these enzymes were detected in increasing numbers of clinical isolates [25,38].

The spread of ESBLs is primarily driven by mutations in the β-lactamase gene, which results in the formation of new, more potent enzymes. These mutations have led to a rapid increase in the prevalence of ESBL-producing bacteria. Among the most prevalent types are the *CTX-M* group, along with *TEM* and *SHV* types. The dissemination of these enzymes occurs mainly through the transfer of plasmids and other mobile genetic elements, facilitating the horizontal spread of resistance across different bacterial strains [24,25].

In the Euregion Meuse-Rhine, notable ESBL types, such as TEM-52, *CTX-M-14*, *SHV-12*, *CTX-M-1*, and *CTX-M-15*, have been identified in *E. coli* isolates. One prominent example of the global spread of ESBLs is the *E. coli* O25:H4-ST131 clone, which is a major cause of urinary tract infections (UTIs). This strain is particularly notable for its ability to acquire new resistance traits, including a high prevalence of *CTX-M-15*, and has been detected across various European countries. The genetic makeup of *E. coli* ST131 strains includes not only ESBL genes, but also resistance to fluoroquinolones, as well as a newly identified carbapenemase gene, which further complicates treatment options for infections caused by this strain [39,40].

## 3. The Role of Human Microbiota in Antibiotic Resistance

The human body contains a great number of living microorganisms in a complicated environment. Antibiotic resistance is one of the mechanisms that has been developed by these microorganisms to survive in these competitive conditions. [41]. In the description of host–bacteria–gene relations, there exist different theories. Firstly, the ‘red queen’ hypothesis, or the ‘arms race’ hypothesis, inspired by Lewis Carroll, suggests that in the hostile and competitive circumstances of the human body, bacteria will employ a number of resistant ways to survive. Resistance genes that are gathered within plasmids, bacteriophages and transposons are the way that these bacteria survive. The Court Jester theory, which suggests that species evolution results from a natural accident, has coexisted with previous theories over time. Meanwhile, Raoult’s recent implication of ‘Alice’s living croquet’ suggests that it is highly unlikely, if not impossible, to predict the behavior of cohabiting organisms in relation to each other [42].

All the theories mentioned, and many others, can accommodate the human microbiota. The cohabitation of multiple organisms is suitable for containing resistance genes and even transferring them, which, at times, can cause the expansion of these resistant and/or virulent genes. According to originally valid research, the environment is an effective factor in the expansion of ARGs. In a defensive act to protect themselves, bacteria develop antibiotic resistance mechanisms. Prior to the industrialization period, the presence of resistance genes in the human microbiome was clinically low. However, with industrialization, antibiotic resistance has become more prevalent, driven by both human activities and the unintended consequences of certain medications [43]. As a result, some gut bacteria have been selected for and have evolved to become highly resistant to antibiotics [41].

Class 1 integrons collected within plasmids and transposons are the main cause of the spread of ARGs. They originally emanated from environmental bacteria in sediments, and they expanded in human microbiota with industrialization [41,44]. After *mer* transposons were identified as responsible for mercury resistance, other compounds were applied, causing bacterial adaptation and the creation of new resistance genes. This also happened with quaternary ammonium and sulfonamides [43]. Recent studies have also found ARGs in the human microbiota in some specific species, such as *Pseudomonas aeruginosa*, *Salmonella enterica*, and *Campylobacter* spp. This highly implies that these recent ARGs could be a serious danger to humans. According to the majority of studies on the effect of antibiotic use on the gut microbiota, antibiotic use is mostly considered to have a reducing effect on the number of microbes in the gut, but an increasing effect on the number of resistance genes. For instance, in research on pre-school children in Finland, macrolides were proven to reduce the number of *Actinobacteria*, and increase the number of *Bacteroides* and *Proteobacteria* [45]. On the contrary, clindamycin decreases the number of *Bacteroides* and basically all kinds of anaerobic bacteria, but increases the number of *Enterobacteriaceae*. [46].

Fluoroquinolones applied in medicine have been proven to cause a selection of fluoroquinolone-resistant strains. [46] Fluoroquinolone-resistant *Staphylococcus* nasal acquisition happens in almost all hospitalized patients (94%), and also in a noticeable proportion of the community (42%). The application of fluoroquinolones in clinical practice has also been effective in causing fluoroquinolone resistance in *E. coli* in the gut. In addition to all the points mentioned, fluoroquinolone resistance is also connected to methicillin resistance, with a fluoroquinolone-resistant *Staphylococcus* co-resistance [47]. Based on research on the intestinal microbiota, as a consequence of the competitive environment between commensal bacteria and pathogens, a mechanism colonization resistance developed. *C. difficile*, *VRE*, *L. monocytogenes*, and *E. coli* are the species that have been shown to effectively enhance colonization resistance against these pathogens [48].

According to recent studies, there is a possibility of a connection between the gut microbiota and the microbiome in the respiratory system in patients dealing with cystic fibrosis. *P. aeruginosa*’s main respiratory colonization occurs shortly after a few adjustments in both microbiotas, which reduces the number of *Parabacteroides* in the gut and increases the abundance of *Salmonella* in the respiratory system [49,50]. The adjustments in the respiratory tract cause bacterial infections to reoccur, and subsequently cause more doses of antibiotic to be applied, which consequently causes multidrug-resistant bacteria; *Staphylococcus aureus* (*S. aureus*), *P. aeruginosa*, and *Haemophillus influenza* are instances of pathogens often found in CF patients’ sputum. The human microbiota is definitely a reservoir for ARGs, and is also a perfectly suitable environment for pathogenic bacteria to acquire more resistance. A great number of factors can affect this microbiota, such as antibiotics, diet, environment, lifestyle, and infections like cystic fibrosis. There is still so much we do not know about antibiotic resistance, one example being what interferes with the transfer between bacteria. A purpose of future studies should be to find out how the human microbiota acquires these ARGs as well as identifying them [51].

## 4. Impact of Antibiotic Overuse in Clinical Settings

The use of antibiotics has been globally expanding, causing antibiotic resistance to become a much more serious and urgent issue. The unnecessary overuse of antibiotics has become a greater risk to patients’ health, as well as being economically insufficient [52]. According to statistics, antibiotic usage by children is measured as high in different countries such as Turkey, China, and even the USA. The pattern of antibiotics being prescribed differs geographically. The amount of non-prescribed antibiotics sold by local pharmacies with unprofessional doctors is measured to be particularly high in LMIC countries such as India. Seasonal differences should also be considered, since antibiotic prescription is at its highest rate in the winter [53]. One of the factors affecting this issue directly is features relating to practitioners. Analyzing their age and the duration of their practice, older practitioners with longer career durations have been proven to prescribe antibiotics more than others [54]. Between 2005 and 2010, antibiotic prescriptions in the United States were generally under control and decreasing among physicians, due to increasing awareness about antibiotic resistance and efforts to promote responsible prescribing. However, during the same period, there was a concerning rise in antibiotic prescriptions by nurse practitioners (NPs) and physician assistants (PAs). This increase may be attributed to several factors, including the growing role of NPs and PAs in primary care and their involvement in patient care decision-making. While these healthcare professionals play a critical role in addressing the demand for healthcare services, the rise in antibiotic prescriptions among them suggests that antibiotic stewardship initiatives may need to be expanded to include all healthcare providers, emphasizing the importance of responsible antibiotic prescribing practices across the board [55].

The fact that the public is awfully misinformed on this subject is clearly observed in different countries. Extensive research in Australia, Italy, Poland, and across Europe has indicated that a noticeable number of people actually believe that antibiotics are effective against the common cold, influenza, or even viral infections [56,57]. Many patients, whether intended or unintended, pressure their physician to prescribe them antibiotics. Although sometimes practitioners can try to convince these patients that antibiotics are not always the necessary solution, nevertheless, knowledgeable practitioners cannot always singlehandedly control these situations; people also need to become more informed on this matter [53,57]. Bacterial infections have been increasing in people, and antibiotics that were being used routinely have lost a noticeable part of their efficacy, causing antibiotic resistance to become a serious health issue [4,58].

Studies led by the Pew Trust have described the creation of 39 new antibiotics, although other studies in this regard have also indicated that the antibiotics currently being used are already stronger than needed [59]. But, 31% of the antibiotics in the process of production have been suggested to be efficient in ESKAPE (*Enterococcus faecium*, *S. aureus*, *Klebsiella pneumonia*, *Acinetobacter baumannii*, *P. aeruginosa*, and *Enterobacter* spp.) infections [4]. The increasing number of new infections as a consequence of antibiotic resistance is one of the most serious challenges in this matter. The expansion of vancomycin-resistant *Enterococcus* (VRE) *faecium* and *Acinetobacter* spp. clones, leading to new infections, are important instances of how dangerous pathogens can be produced from a simple innocuous GI bacteria [60].

Surgeries and chemotherapies have been affected intensely by the escalation of antibiotic resistance. According to research conducted in the USA, many pathogens lead to infections in surgical processes and after chemotherapy processes [61]. These infections appear to be resistant to routine antibiotic treatments. Furthermore, several previously effective treatments have become less viable due to the growing issue of antibiotic resistance. In particular, the colonization of carbapenem-resistant *Enterobacterales* (CRE) has emerged as a significant concern, contributing to a notable incidence of infections among patients undergoing stem cell transplantation (SCT), thereby complicating their clinical management and recovery [60]. The optimized and efficient use of antibiotics is also a challenge in intensive care units. For instance, bacterial sepsis or systemic inflammatory response syndrome are conditions that could result in the overlap of serious clinical syndromes [62] (Table 1).

### Hospital-Acquired Infections (HAIs)

Infections acquired in healthcare settings, such as hospitals, exceed 1.7 million cases each year, leading to almost 99,000 deaths. ESKAPE pathogens, which include *Enterococcus faecium*, *S. aureus*, *Klebsiella pneumoniae*, *A. baumannii*, *P. aeruginosa*, and *Enterobacter* spp., are the key factors contributing to these circumstances [24,63]. These play a major role in increasing healthcare costs and economic challenges, and are frequently described as unresponsive to various medications. Among the last four of these, KAPE, are bacteria belonging to the *Proteobacteria* phylum, which accounts for 43% of all bacterial pathogens, the highest percentage compared to other phyla [25,36]. It is crucial to focus on antibiotic resistance in harmful *Proteobacteria*, and finding new ways to combat MDR bacteria is a top concern. According to the World Health Organization (WHO), the primary focus for new treatment research and development lies in addressing the top three microbial infections, which include *A. baumannii*, *P. aeruginosa*, and carbapenem-resistant ESBL-producing *Enterobacteriaceae*, all belonging to the KAPE *Proteobacteria* group [64]. Infections that are resistant to various antibiotics, including other β-lactams, are typically treated with carbapenems. β-lactam antibiotics are extensively used across the world, and β-lactamases are among the most prevalent and well-researched antimicrobial resistance gene categories [65].

These resistance genes are commonly located on mobile genetic elements (MGEs), which play a crucial role in amplifying the burden of dietary reference intakes. One of the most commonly recommended antibiotics on a global scale is amoxicillin, an aminopenicillin, which is primarily used to combat otitis media ear infections [21,35]. *E. coli*, a type of Gram-negative *Proteobacteria*, is a common cause of these infections that lead doctors in the USA to frequently prescribe antibiotics. In the present day, up to 95% of *E. coli* strains stored in national repositories exhibit amoxicillin resistance. *CTX-M* genes, known for their high mobility and prevalence in various isolates, are the predominant and significant types of extended-spectrum β-lactam (ESBL) enzymes, despite the wide array of enzymes that provide ESBL resistance. ESBLs were first identified as enzymes with the ability to hydrolyze the β-lactam ring in nearly all β-lactam categories, with the exception of carbapenems [24,25,39,66].

**Table 1:** Impacts of antibiotic resistance mechanisms in bacteria and their implications for treatment.

Mechanism	Description	Examples	Pathogens Involved	Implications for Treatment	Epidemiological Relevance	Ref.
Genetic Mutations	Spontaneous changes in bacterial DNA that lead to resistance	Mutations in gyrA/gyrB (fluoroquinolone resistance), RNA polymerase gene	*M. tuberculosis* *E. coli* *P. aeruginosa*	Loss of efficacy of existing drugs, requiring alternative therapies	High prevalence in nosocomial infections and community settings	[67]
Horizontal Gene Transfer	Transfer of resistance genes between bacteria via conjugation, transduction, or transformation	*blaNDM*-1 gene spread (carbapenem resistance)	*Enterobacteriaceae*	Rapid spread of resistance, limited treatment options	Global dissemination, especially in regions with poor antibiotic regulation	[68]
Efflux Pumps	Proteins that expel antibiotics out of bacterial cells, reducing intracellular drug concentrations	MexAB-OprM in *P. aeruginosa*	*P. aeruginosa* *A. baumannii*	MDR across antibiotic classes	Significant in ICU and immunocompromised patients.	[69]
Biofilm Formation	Formation of protective extracellular matrices by bacterial communities, hindering drug penetration and immune response	Biofilms in *P. aeruginosa*, *S. aureus*	*P aeruginosa*, *S. aureus*	Chronic infections, limited response to treatment	Common in medical devices and chronic infections like CF	[70]
Intrinsic Resistance	Natural resistance inherent to specific bacterial species without prior exposure to antibiotics	*E. coli* resistance to vancomycin	*E. coli* *P. aeruginosa*	Infeasibility of certain treatments from the outset	Universally present, but varies across species and environments	[71]
Stress Response Mechanisms	Synthesis of survival molecules like (p)ppGpp during stress, activating defense pathways and reducing metabolic activity	Observed in nutrient deprivation or antibiotic exposure	*P. aeruginosa* *E. coli*	↑persistence under adverse conditions	Contributes to resilience in hospital settings	[72]
Target Site Alteration	Structural changes in the antibiotic binding site, reducing drug affinity and efficacy	Mutations in 23S rRNA gene (macrolide resistance)	*M. tuberculosis* *K. pneumoniae*	Resistance to specific drug classes, such as macrolides	Widespread in pathogens with high mutation rates	[73]
Antibiotic Misuse and Overuse	Overprescription and use of antibiotics in clinical and agricultural settings	MRSA, CRE	*MRSA* *E. coli* *P. aeruginosa*	↑healthcare costs, ↑hospital stays	Major driver of resistance worldwide, particularly in LMICs	[74]
Selection Pressure	Environmental and clinical use of antimicrobials, creating a survival advantage for resistant bacteria	*K. pneumoniae* under carbapenem use	*K. pneumoniae* *P. aeruginosa*	↑emergence of MDR strains	Notable in regions with high antibiotic consumption	[75]
Plasmid-mediated Resistance	Spread of mobile genetic elements carrying resistance genes	*CTX-M-15* plasmid-mediated resistance in *E. coli*	*E. coli* *K. pneumoniae*, *Salmonella* spp.	Accelerated global dissemination of resistance traits	Prominent in hospital outbreaks and agricultural use settings.	[76]
Environmental and Industrial Impact	Exposure to pollutants or heavy metals, driving bacterial adaptation and resistance	Resistance to mercury and QACs	*P. aeruginosa* *Salmonella enterica*	↑ARG prevalence in environmental reservoirs	High relevance in industrialized and polluted areas	[77]
Impact on Gut Microbiota	Alteration in gut microbial diversity due to antibiotic exposure, promoting ARG reservoirs	↓*Actinobacteria* ↑*Proteobacteria*.	*Bacteroides* spp., *Enterobacteriaceae*, *Clostridium difficile*	↑risk of secondary infections and microbiota imbalance	Significant in patients with recurrent antibiotic treatments	[78]
Adaptive Metabolism	Bacteria reduce growth or switch to alternative pathways under antibiotic stress	Use of alternative enzymes in *E. coli* under trimethoprim treatment	*E. coli* *Salmonella* spp.	↓antibiotic efficacy in targeting active metabolism	Observed in long-term infections and biofilm-associated bacteria	[79]

↑: Increase; ↓: Decrease.

## 5. Antibiotic Resistance in Healthcare: Challenges in Treating Common Infections

Instances of antibiotic-resistant infections in clinical scenarios have been linked to agricultural animals, with drug-resistant *Salmonella* infections from poultry being a notable example dating back to the 1960s [80]. The theory known as the “farm-to-fork” concept suggests that antibiotic-resistant bacteria present in livestock can be transmitted to humans via direct interaction or through tainted animal goods. If these bacteria that are resistant to antibiotics invade the GI tract of a human, they might result in an infection that needs to be treated with antibiotics, but the primary medication may not be successful [80]. Initially discovered in bacteria linked to animals, various significant ARGs have now transferred to strains associated with humans, such as the *mcr-1* colistin resistance gene found in colistin-resistant *E. coli* from pigs. It is important to regulate and decrease the use of antibiotics in farm animals to the minimum required for veterinary purposes [28]. The ban on antibiotics as growth promoters in agriculture is in place in countries like the Netherlands, Denmark, Sweden, and the UK [81]. On the other hand, some nations (like China) are still employing antibiotics that are vital for human health, such as the final-resort antibiotic colistin, for this specific purpose. It is clear that further steps must be taken to reduce the inappropriate use of antibiotics in humans and animals [64,82].

### 5.1. Impact of Resistant Infections on Hospital Stay Duration

Resistant infections are frequently associated with prolonged hospital stays, which are often used as a proxy for morbidity. Infections caused by resistant pathogens, such as Methicillin-resistant *S. aureus* (MRSA), ESBL-producing *Klebsiella* spp., *E. coli*, and penicillin-resistant *pneumococci*, are well-documented to extend the length of hospital admissions [83]. MRSA, for example, is a major cause of surgical site infections and can significantly prolong hospital stays, with studies indicating that patients with MRSA infections may experience hospitalizations lasting several days longer than those with susceptible strains. Similarly, patients infected with penicillin-resistant *pneumococci* tend to have extended hospital stays compared to those with penicillin-susceptible strains, due to the need for alternative, more intensive treatments. The duration of hospitalization is also markedly increased for individuals infected with ESBL-producing organisms such as *Klebsiella* spp. and *E. coli*, as these infections are more difficult to treat with conventional antibiotics, often necessitating the use of broader-spectrum or combination antibiotics. This extended stay not only increases the burden on hospital resources, but also exacerbates patient morbidity, as prolonged exposure to the hospital environment increases the risk of additional healthcare-associated infections and complications. Moreover, the delayed recovery resulting from resistant infections can further compound the length of stay, with patients requiring additional surgeries, prolonged antibiotic courses, and intensive care [81,83].

### 5.2. Cost Implications of Antimicrobial Resistance

Antimicrobial resistance (AMR) has significant cost implications for healthcare systems worldwide, primarily due to the increased expenses associated with managing resistant infections. These costs stem from multiple factors, including the use of more expensive alternative medications, extended hospital stays, additional diagnostic tests, and the need for more intensive and specialized care. Patients infected with resistant bacteria, such as penicillin-resistant *pneumococci*, MRSA, and ESBL-producing *E. coli* and *Klebsiella* spp., often require complex treatment regimens. This reliance on second-line or last-resort antibiotics, which are generally more costly than standard therapies, significantly drives up the cost of care [81,83].

Moreover, the longer duration of hospitalization associated with resistant infections exacerbates these costs. For example, infections caused by MRSA or ESBL-producing organisms frequently necessitate prolonged monitoring, additional interventions such as repeat surgeries, and extended antibiotic therapy, all of which contribute to increased healthcare expenditure [84]. Studies have demonstrated that the financial burden of treating patients with resistant infections can be several times higher than that of managing infections caused by susceptible strains. Additionally, indirect costs, such as lost productivity due to extended illness or time away from work, further amplify the economic impact. The burden of AMR also extends to healthcare infrastructure, as hospitals and clinics must allocate additional resources to infection control measures, staff training, and the implementation of antimicrobial stewardship programs. The financial strain caused by AMR highlights the urgent need for comprehensive strategies to curb its spread. Investments in rapid diagnostic tools, novel antimicrobial development, and global collaboration to implement effective antibiotic stewardship are essential to mitigate the rising costs associated with resistant infections and ensure sustainable healthcare delivery [81].

### 5.3. Impact of Antimicrobial Resistance on Mortality in Enterococcal and S. aureus Infections

*Enterococci*, among other highly resistant bacteria, tend to have low levels of virulence and mainly affect hospitalized patients with severe underlying illnesses and/or weakened immune systems. In such instances, distinguishing whether the resistance or the underlying conditions are to blame for the adverse outcome may be complex. However, research conducted both before and after the event has indicated a higher likelihood of mortality in cases of enterococcal infections caused by VRE, with two of these studies comparing patients without such infections as a reference group [81,83]. Analysis of various research on *S. aureus* bacteremia indicates that patients with MRSA have an elevated risk of mortality when contrasted with individuals with MSSA. Hemodialysis patients with *S. aureus* bacteremia face a greater likelihood of death if infected with MRSA rather than MSSA, as indicated by a prospective study. Likewise, there is a correlation between MRSA surgical site infections and an increased likelihood of a fatal outcome in studies [81,85].

### 5.4. Selection Pressure and Risk Factors

It is probable that the mold on Fleming’s agar plates evolved penicillin as a way to survive in a natural habitat in which it competes with many other organisms. Bacteria create defenses to combat harmful substances, whether they come from nature or humans, in much the same way. Antimicrobial resistance is an inherent process that aids microorganisms in their survival amidst harmful substances in their surroundings [81,85]. Without the presence of the toxic substance, bacteria with antimicrobial resistance mechanisms may experience a disadvantage. Bacteria with resistance mechanisms thrive in environments with antimicrobials, like hospitals, through Darwinian selection. Therefore, the utilization of antimicrobial drugs, whether correct or not, may cause the development of resistant bacteria. The contrast between Southern and Northern European countries is evident in their higher rates of outpatient antimicrobial consumption and antimicrobial resistance [85].

**Figure 4:**
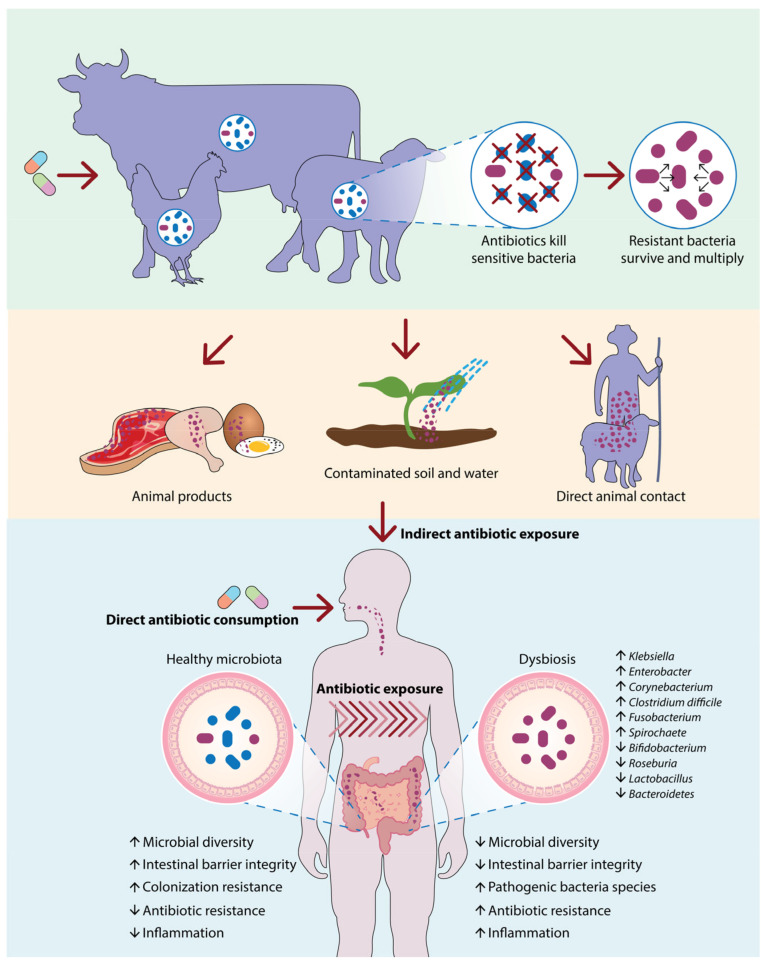
Pathways of antibiotic resistance transmission and its impact on the human microbiome. The diagram illustrates the transmission of antibiotic resistance through livestock, the environment, and human consumption. Antibiotic use in animals fosters resistant bacteria, which can contaminate the environment or enter the food chain. Human exposure, both direct and indirect, contributes to dysbiosis, disrupting microbial diversity and intestinal integrity, and increasing inflammation, ultimately promoting antibiotic resistance and pathogenic bacterial growth.

Beyond the application of antimicrobials, there are other factors that have been singled out as risk elements for developing infections with resistant bacteria. Factors that increase the chances of acquiring infections from ESBL-producing bacteria in hospitals include being admitted to the ICU, receiving parenteral nutrition, having indwelling catheters, suffering from renal failure, and experiencing burns [39,86]. Factors that increase the likelihood of acquiring infections from ESBL-producing bacteria in community settings include recent use of antimicrobial medications, especially cephalosporins, being over 60 years old, having diabetes, and recent hospital stays. The current rise in antimicrobial drug resistance worldwide might be influenced by the HIV epidemic [25] (Figure 4).

## 6. Strategies for Combating Antibiotic Resistance: New Drugs and Alternative Therapies

Antibiotics have significantly contributed to the preservation of patients’ lives and have been instrumental in facilitating numerous advancements in the fields of medicine and surgery. The prevalent use of antibiotics has given rise to a serious challenge referred to as “antibiotic resistance.” This phenomenon primarily occurs due to the over prescription and incorrect application of antibiotics, which fosters the growth of bacteria that cannot be treated effectively with these drugs. To put it differently, these microorganisms have developed a “resistance” to antibiotic treatments [66]. An effective stewardship initiative can be categorized into a three-tiered structure. The initial level comprises the critical structural or systemic prerequisites that a medical institution needs to meet in order to successfully implement stewardship in a hospital. This comprises, for example, the stewardship panel noted before, an infrastructure designed to monitor antibiotic application, and the availability of localized directives [66].

The central tier outlines possible goals for stewardship initiatives. The stewardship program and its team center on these elements of proper antibiotic utilization. Hospital stewardship groups ensure the responsible use of antibiotics by regularly assessing their stewardship goals and implementing strategies for enhancement when needed to optimize usage. Evaluating and overseeing the actions of A-teams focused on improving the correct use of antibiotics necessitates the ability to measure how well antibiotics are being utilized [66,87]. Consequently, global specialists have employed a modified Delphi method from RAND to develop a collection of quality metrics aimed at establishing and assessing the appropriateness of antibiotic usage for treating all bacterial infections in hospitalized adults. This collection, which forms the foundation of the second tier of the pyramid, outlines the recommended care at the individual patient level, and will be referred to as stewardship goals [66].

### Antimicrobial Stewardship

The choice of antibiotics, their dosage, and the route of administration are critical factors influencing antibiotic efficacy and resistance development. In the United States, up to 50% of antibiotic consumption is considered excessive, contributing significantly to resistance. Overuse stems from various factors, including prescribing antibiotics without sufficient evidence of bacterial infection, treating suspected non-bacterial infections, and a lack of stringent regulations requiring a prescription for antibiotic purchase. The ease of access and patient demand also exacerbate misuse [66]. Defined daily doses (DDDs) serve as a standardized metric to evaluate antibiotic usage, reflecting the average daily dose for adults for a drug’s primary administration. For example, the recommended DDD for amoxicillin is 1.5 g per day administered orally. Analyzing DDDs allows for the assessment of antibiotic usage trends at the hospital or national levels. In 2015, the United Kingdom reported an average antibiotic consumption rate of 8696 DDDs per 1000 individuals per day, translating to 8696 DDDs per individual daily. Unlike some other countries, antibiotics in the UK are strictly available by prescription from licensed professionals, and national initiatives to reduce their inappropriate use have been pivotal in curbing resistance [66,88].

Turkey, by contrast, has one of the highest global rates of antibiotic consumption, recorded at 18,095 DDDs per 1000 individuals daily. Factors contributing to this elevated usage include insufficient medical education on antibiotic stewardship, aggressive marketing by pharmaceutical companies, and inadequate regulatory frameworks. Amoxicillin remains the most widely consumed antibiotic in Turkey, reflecting its widespread application and the challenges associated with regulating broad-spectrum antibiotics. Nevertheless, Turkey has made strides in addressing overuse through campaigns aimed at raising awareness of antimicrobial resistance and the implementation of policies to control antibiotic sales and prescriptions. The choice of antibiotic—whether narrow-spectrum or broad-spectrum—plays a pivotal role in determining its impact on resistance development. Broad-spectrum agents, like amoxicillin, are often overprescribed, even when narrower-spectrum alternatives might suffice. Similarly, inappropriate dosage—whether too low or excessively high—can either fail to eradicate the pathogen or promote resistance. The route of administration, whether oral, intravenous, or intramuscular, also influences treatment outcomes. Intravenous antibiotics, for instance, are typically reserved for severe infections, but their misuse in non-critical cases can unnecessarily increase resistance risks. In brief, the interplay of antibiotic selection, dosage, and administration underscores the complexity of combating resistance. Countries like the UK have shown progress through strict prescription policies and stewardship programs, while high-consuming nations such as Turkey highlight the urgent need for global action. Addressing these variables comprehensively is essential to reducing resistance rates and preserving antibiotic efficacy worldwide [66,88].

The emergence of antibiotic resistance can be traced back to the early days of antibiotic discovery. A key factor contributing to the rapid rise of antibiotic resistance is the ability of bacteria to exchange genes instantly with one another, facilitating the sharing of resistance. In hospitals, the occurrence of resistant staphylococcus infections surged by 14% from 1946 to 1948. Upon discovering bacteria that could survive penicillin treatment, scientists worked on creating methicillin, a new antibiotic designed to fight these resistant strains. Initially, methicillin showed promise in treating infections that did not respond to penicillin, but it was not long before methicillin-resistant strains, referred to as MRSA, emerged [32].

The development of β-lactams, a distinct group of antibiotics, has emerged as a promising solution for addressing the challenge posed by antibiotic-resistant bacterial strains. The application of these β-lactams in clinical practice has produced successful results. In both the UK and the USA, cases of infections resistant to β-lactam treatments were quickly found. The introduction of vancomycin in 1972 marked the arrival of a new antibiotic aimed at treating infections that were resistant to earlier antibiotic treatments [24,32]. Early on, it was considered that the formation of resistance to vancomycin would be a significant hurdle, but by 1979, there were documented cases of infections that had developed resistance to it. Between 1950 and the 1970s, a variety of novel antibiotics emerged to combat infections such as endocarditis, plague, respiratory illnesses, and meningitis. The rapid availability of the latest antibiotics to the general population eventually set the stage for the development of resistance to these drugs. Over time, certain bacterial strains developed resistance to various antibiotics, leading to the emergence of MDR infections caused by these pathogens [63,89].

## 7. Future Perspectives: The Role of Policy, Research, and Public Awareness in Mitigating Antibiotic Resistance

### 7.1. Future Perspectives

Addressing antimicrobial resistance (AMR) requires a unified One-Health strategy and the adoption of innovative technologies to explore various ecological environments, thereby reducing the risk of transmission among humans, animals, and their shared surroundings. Next-generation sequencing, when applied in a One-Health context, is a powerful tool for analyzing the dissemination of antimicrobial resistance and the emergence of resistant infectious agents. While Illumina is currently the predominant sequencing technology, ONT sequencing is progressing swiftly, and its latest chemical advancements allow for more than 99% accuracy in raw reads alongside substantial data output [24,63,90]. Furthermore, Oxford Nanopore Technologies (ONT) sequencing offers opportunities for various groundbreaking sequencing techniques, including immediate data analysis and field-based sequencing. The introduction of “Infinity”, a new long-read technology, by Illumina in January 2022, brought together the reliability of Illumina sequencing and the benefits associated with long-read methodologies. In hospitals, environmental sampling typically occurs solely during investigations of outbreaks, and only when the infection control team is motivated and equipped for this type of analysis [24,90,91].

The primary objective of antimicrobial stewardship teams is to maintain high standards in antibiotic application by systematically evaluating and improving a set of stewardship targets. These targets can encompass actions such as refining empirical therapy to align with specific pathogens once culture results are obtained, or converting intravenous treatments to oral forms after 48 to 72 h. Achieving optimal performance levels for these quality metrics is viewed as adhering to the goals of antibiotic stewardship. Nonetheless, supporting data for every specific stewardship goal have not been evaluated so far. This situation obscures the potential outcomes of refining one particular aim at the patient level, making it challenging for local oversight teams to identify where to concentrate their enhancement strategies [66,87].

### 7.2. General Public

It is important to consider the broader audience and methods to engage with them effectively. It is essential to engage the community in initiatives aimed at enhancing the effectiveness of antibiotic usage. It appears that a universal strategy is impractical, as nearly 4 million individuals in the Netherlands are first- or second-generation immigrants, representing around 200 different ethnic groups [24]. Research indicates that migrants in Europe exhibit a greater incidence of antimicrobial resistance, which may be linked to increased antibiotic consumption among individuals from non-Dutch backgrounds. This occurrence could be attributed to variations in the understanding of antibiotics or differing practices among physicians when prescribing for various groups of patients. To the best of our understanding, there has been no research conducted on the connection between antibiotic usage and awareness of antibiotics across various ethnic communities. Clarifying this matter could open up fresh avenues for healthcare policymakers to tailor their initiatives more effectively [24].

### 7.3. Hospital Setting

There is a push to refrain from utilizing broad-spectrum or emergency antibiotics to promote the responsible application of these medications. Some studies have shown that by enforcing limitations on the use of certain antibiotics, either through specialist preapproval, or by permitting their use for just 72 h with subsequent authorization needed for extended use, there can be a decrease in costs and a lower prevalence of MDR bacteria. Restricting the use of certain classes of antibiotics is widely regarded as an effective strategy for improving antimicrobial stewardship by reducing the selection pressure that drives resistance. However, the overall impact of such restrictions remains uncertain, as they can inadvertently lead to a compensatory increase in the utilization of other, unrestricted antibiotics. This shift in usage may result in elevated resistance levels to these alternative antibiotics, potentially undermining the intended benefits of the restriction and complicating efforts to control antimicrobial resistance on a broader scale [66].

### 7.4. Role of Policy

Policy plays an indispensable role in addressing the multifaceted challenge of AMR. A robust policy framework is essential for the effective implementation of One-Health strategies, which integrate human, animal, and environmental health to combat AMR comprehensively. Policies can facilitate the adoption of innovative technologies, such as NGS and ONT, by providing funding, setting research priorities, and ensuring equitable access to these advancements. For example, policy-driven initiatives can incentivize hospitals and research institutions to utilize sequencing technologies for environmental sampling and outbreak investigations, thereby enhancing surveillance systems [92].

Antimicrobial stewardship programs in healthcare settings also require policy-driven support to achieve their objectives. Policymakers must prioritize the development of evidence-based guidelines that target key stewardship practices, such as optimizing empirical therapy, transitioning from intravenous to oral antibiotics, and monitoring the effectiveness of stewardship interventions. Policies can further mandate the regular evaluation of stewardship goals and their patient-level outcomes to ensure the refinement of these programs based on reliable data. Without such policy guidance, local oversight teams may struggle to identify impactful strategies, thereby limiting the success of stewardship efforts. On a broader scale, public health policies must address disparities in antibiotic usage and awareness among diverse ethnic and immigrant communities. Tailored educational campaigns, informed by community-specific research, can be implemented to enhance public understanding of antibiotics and promote their responsible use [93].

Finally, within hospital settings, policy mechanisms can regulate the use of broad-spectrum antibiotics by enforcing restrictions, such as preapproval requirements or time-limited authorizations. However, these policies must be carefully balanced to avoid unintended consequences, such as increased reliance on alternative antibiotic classes and the subsequent rise in resistance. Comprehensive policymaking is essential to ensure that stewardship interventions are both effective and adaptable, mitigating the broader implications of AMR while maintaining optimal patient care outcomes. By fostering collaboration among policymakers, researchers, healthcare professionals, and the public, a well-structured policy framework can address the key challenges in AMR mitigation and pave the way for sustainable solutions [94].

## 8. Conclusions

Antibiotic resistance has undermined decades of medical advancement and grown into a serious worldwide health concern. Resistance is driven by a variety of processes, including biofilm development, horizontal gene transfer, and genetic changes. These methods underscore the complexity of this problem. Urgent action is required due to the clinical and economic consequences that have been highlighted by the increase in multidrug-resistant bacteria. Resistant pathogens, including multidrug-resistant organisms, not only compromise treatment outcomes, but also impose severe economic and clinical burdens, such as prolonged hospital stays, increased healthcare costs, and heightened mortality rates. Action to mitigate this includes enhancing antimicrobial stewardship programs, advancing public education to promote responsible antibiotic use, and implementing rigorous global policies to regulate antibiotic consumption. A unified global effort, rooted in innovation, collaboration, and sustainability, is imperative to preserve the efficacy of existing antibiotics and to foster the development of novel therapeutic solutions that are capable of combating this pressing health threat.

## Data Availability

The corresponding authors will provide the datasets created during and/or analyzed during the current investigation upon reasonable request.
